# Effect of a Mobile App on Medication Errors During Simulated Pediatric Cardiopulmonary Resuscitation: PEDIDOSIS App vs. Conventional Methods. A Multicenter Simulation-based Randomized Controlled Trial

**DOI:** 10.1007/s10916-026-02425-x

**Published:** 2026-06-19

**Authors:** Ana Martínez-Serrano, Héctor Ruiz-Rojo, Margarita Ruano Encinar, Paula García-Sánchez, Carlos Durantez-Fernández, Teresa de Rojas

**Affiliations:** 1 Pediatric Emergency Department, La Paz University Hospital, Madrid, 28029 Spain; 2 Department of Nursing, Nursing School of Palencia, University of Valladolid, Palencia, 34004 Spain; 3 Department of Nursing, Faculty of Nursing, University of Valladolid, Valladolid, 47003 Spain; 4 Arztpraxis Mylife, Fulenbach, Solothurn, 4629 Switzerland

**Keywords:** Administration, Intravenous, Emergencies, Medication Errors, mHealth, Nursing, Pediatrics

## Abstract

Despite its low incidence, pediatric cardiopulmonary arrest remains associated with high mortality and morbidity. The adequate preparation and administration of drugs can impact favourably on the outcomes of cardiopulmonary resuscitation. Nursing staff rely on cognitive aids such as paper support, calculators, or spreadsheets to ensure correct drug administration. Numerous strategies have been proposed to prevent medication errors, including the emergence of mHealth. This study evaluates the efficacy and usefulness of the PEDIDOSIS smartphone application (app) for nursing staff in a simulated pediatric emergency setting. A multicentre high-fidelity simulation-based randomized controlled trial was conducted in a pediatric emergency setting with nursing professionals from different hospitals in Spain. Randomisation groups: PEDIDOSIS app (La Paz Hospital, Spain) vs. conventional methods (such as printed or electronic medication tables, smartphones, calculators or no cognitive aid). To avoid bias due to differences in pediatric emergency experience, each group was randomly assigned the same number of participants with experience (> 5 years) and without experience (≤ 5 years). Epinephrine, bicarbonate, midazolam, and norepinephrine on two ocassions were administered in a simulation scenario. Number and magnitude of medication errors, time taken to drug administration were evaluated as measured with video recording; stress, satisfaction, and usability as measured using standardized survey tools were evaluated. One-hundred and five nurses participated. The incidence of wrong dose administration by any deviation from the correct dose was 51.5% for the control group vs. 26.4% for the PEDIDOSIS app group (p < 0.001). The OR for a wrong dose administration > 10% deviation with PEDIDOSIS app vs. conventional methods was 0.24 [0.14–0.4]), with a risk difference of 20.7%. The proportion of participants committing several medication errors (ME) was substantially lower with the app (15.1%) than with conventional methods (57.7%). Overall satisfaction regarding drug preparation was higher and stress lower in the app group. The System Usability Scale (SUS) score obtained for PEDIDOSIS app was 78.9 out of 100.

## Introduction

Pediatric cardiopulmonary arrest, despite its low incidence, is associated with high mortality and severe long-term sequelae [1]. During the first 15 min of cardiopulmonary resuscitation (CPR), survival and favourable neurological outcomes decreased linearly by 2.1% and 1.2% per minute, respectively [2, 3]. Recent studies report an in-hospital survival rate of approximately 38% following pediatric in-hospital CPR, whereas survival after out-of-hospital pediatric CPR ranges from 6% to 20% [4, 5].

Medication errors (ME) are among the leading causes of preventable harm in healthcare worldwide [6, 7]. According to the National Coordinating Council for Medication Error Reporting and Prevention (NCC MERP), ME are defined as preventable incidents that may cause harm to the patient or lead to the improper use of medications [8]. One specific category of ME is incorrect preparation, which occurs when a medication is improperly formulated or manipulated before being dispensed or administered [9]. Children are considered a particularly high-risk population for severe ME, leading international health organizations to prioritize the implementation of strategies aimed at reducing these errors in pediatric care [10]. Within pediatric CPR care, appropriate drug preparation and administration may positively influence clinical outcomes, underscoring the importance of early drug delivery [2, 3].

While many medications can be administered without dilution as direct intravenous drugs (bolus), others require complex calculations during preparation and dilution prior to administration. Eliminating the need for drug dilution has been identified as a relevant strategy to reduce pediatric ME [11] and should be avoided whenever possible [12]. ME have been reported more frequently with drugs requiring dilution compared with bolus medications [6]. When dilution is unavoidable, preparation instructions must be clear and precise [12].

Despite pediatric emergency services being specialized and adequately prepared for CPR, numerous ME have been reported during pediatric CPR simulations [12]. Certain studies conducted in simulated scenarios have documented intravenous ME in more than 41% of cases, with 65% attributed to incorrect dose, the most common type of error. Other simulation studies have reported dosing error rates ranging from 26% to 70% [2, 4, 13], and in one study, ME were identified in 7 out of 10 simulations [6].

In recent years, multiple Mobile Health (mHealth) innovations have been developed to enhance safety in drug administration [12]. Several studies have demonstrated that the use of mobile applications in pediatric emergency settings is associated with a reduction in ME [1, 3, 5, 13–18]. Among healthcare professionals, mobile applications have become an integral part of clinical practice and have contributed to transforming healthcare delivery [19].

In this context, in 2023, three pediatricians, one pharmacist, two pediatric nurses of La Paz Hospital (Madrid, Spain), and one Information Technology specialist in our team started and created PEDIDOSIS app (La Paz Hospital, Spain). It’s a team with proven experience of over 15 years. It was completely developed in 2024 [20]. Aware of advances in mHealth, technology related to pediatric patient safety has been developed, in line with what is being done in other European countries [2, 14, 15]. The app was designed and validated by a multidisciplinary team comprising pediatricians, nurses, and a pharmacist with robust evidence [21]; In the beginning, it was developed in Spanish. PEDIDOSIS app was developed to provide technology adapted to healthcare professionals, their frontline workflows, and ease of use, which constitutes its primary objective. The stress and workload required by the clinical app might differ from those of conventional methods.

### App Features

The PEDIDOSIS app prototype was tested by five intensive care nurses through an internal pilot study and was described as intuitive and easy to use.

After patient age and weight input, the app provides recommendations for appropriate preparation and administration of intravenous emergency drugs. Its main purpose is to ensure accurate and quick information access, particularly relating to emergency drugs for children. To calculate a dose using the PEDIDOSIS app, the user must enter the patient’s weight or age, and then select or search the drug(s) listed. The clinical app automatically calculates the doses based on weight (values are derived from a literature review made by a multidisciplinary team [22–26]). The calculated dose, its preparation and administration are displayed on the screen. In contrast, using conventional methods (such as printed or electronic medication tables, smartphones, calculators or no cognitive aid) requires multiple calculations like weight multiplied by medication dose or divided by vial content.

The administration steps are displayed on the app in a simplified manner:

- Bolus: 1-Dose (IV); 2-Amount taken from the vial/ampoule; 3-Dilution; 4-Administration time.
- Infusion: 1-Infusion dose (IV); 2-Serum to be used; 3-Amount taken from the vial/ampoule; 4-Dilution; 5-Infusion administration time.

**Fig. 1:**
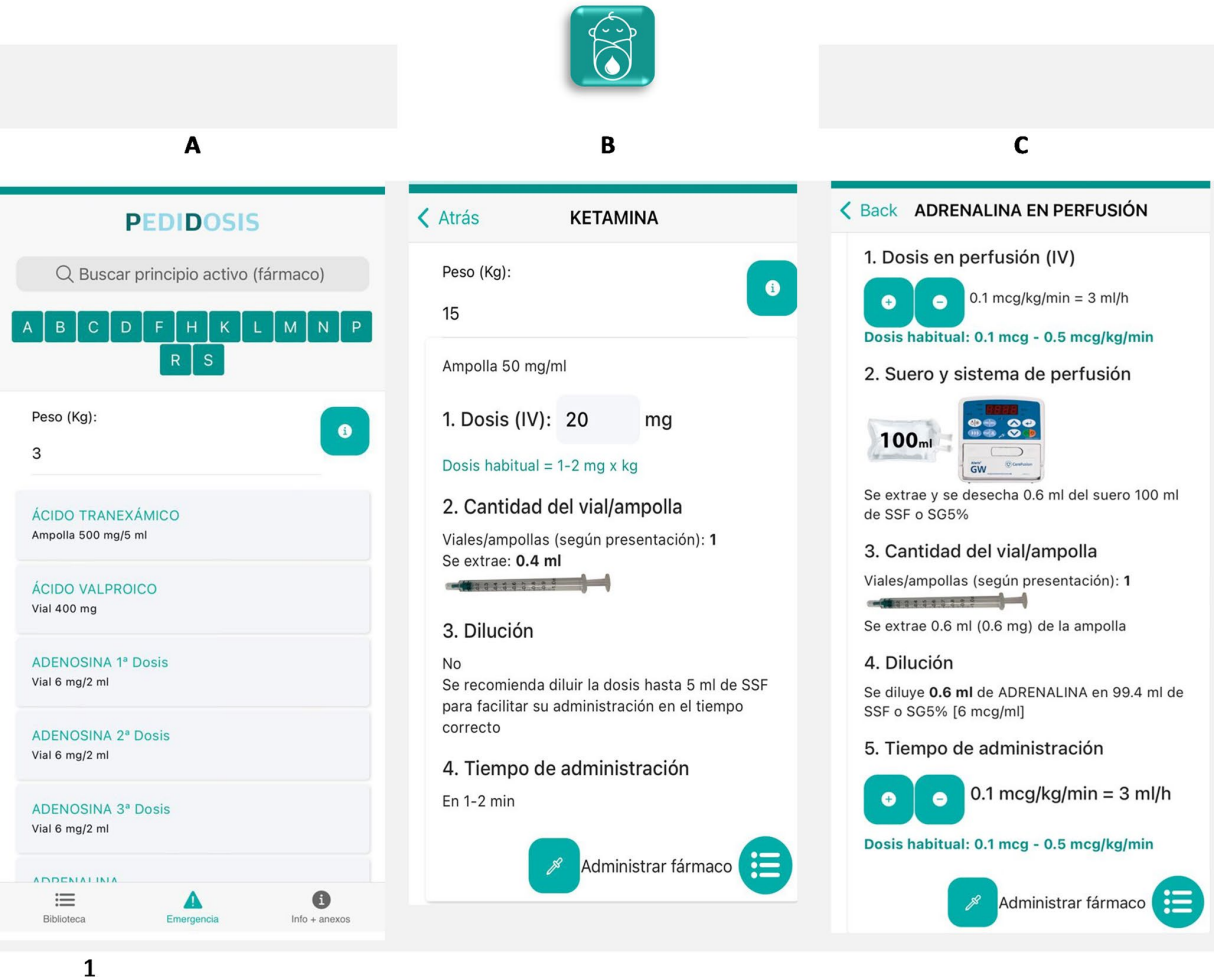
Screenshots of the PEDIDOSIS mHealth app: image **A** is the home screen where the user can search or select a specific drug, **B** is the screen how one intravenous drug (ketamine) is displayed, **C** is the screen how one perfusion drug (epinephrine) is displayed

For each drug, the exact amount needed to prepare the drug is clearly displayed, eliminating the need for calculations. The app automatically calculates the data related to the preparation and administration of the drug sequentially (extraction of the precise amount from the vial/ampoule, amount of dilution required, administration time, etc.) based on the weight entered. The drugs are displayed in alphabetical order with the drug preparation amounts adapted to the patient’s weight. The app has an interactive screen, allowing to modify the patient’s weight, drug dose, select several drugs at once and confirm drugs administered; in the case of infusions, increase or decrease the rate, pause, end the infusion, etc. The app can also be used offline (Fig. 1 shows screenshots of the PEDIDOSIS app). All the content related to the PEDIDOSIS app can be accessed via a cross-platform application: https://pedidosis.app/ [20]; with the iOS operating system [27] and with the Android operating system [28]. Currently, access to the application is restricted to healthcare professionals and researchers as a research use only (RUO) app while its formal regulatory classification is under review (March 2026) by the Spanish Agency of Medicines and Medical Devices (AEMPS), in accordance with the criteria established under Regulation (EU) 2017/745 on medical devices.

The objective of this study is to assess the efficacy of PEDIDOSIS app in reducing the occurrence of ME compared with conventional methods during simulated pediatric CPR scenario.

## Methods

### Study Design and Setting

A multicentre high-fidelity simulation-based randomized controlled trial was conducted at six hospitals in Spain (La Paz, Madrid; Virgen de la Arrixaca, Murcia; Infanta Cristina, Parla; Niño Jesús, Madrid; Infanta Sofía, San Sebastián de los Reyes; Río Carrión, Palencia). This study was approved by the Internal Scientific Committee of the Health Research Institute of La Paz University Hospital (IdiPAZ) in March 2024 (HULP code: PI-6112). This study followed the Consolidated Standards of Reporting Trials (CONSORT) reporting guideline [29]. Data collection took place between April 2024 and June 2024.

Videos of the participants’ administration focused only on the hands of each participant to ensure anonymity. The videos generated from this recording were stored in an encrypted and restricted access folder, which was only accessed by the study researchers for data analysis. They were subsequently destroyed.

### Participants

The participants were nurses or pediatric nursing residents working in a pediatric emergency department of one of the six participating hospitals. Participation was voluntary and each participant approached during their working hours. The intervention was performed an in-situ simulation. The exclusion criterion was unwillingness to participate or failure to complete the intervention. No incentives were provided.

### Randomisation

A stratified randomisation was performed using REDCap (Research Electronic Data Capture, United States) [30, 31] with a1:1 ratio into two groups: the app group and control group. To prevent differences in pediatric emergency experience from introducing bias, each group was randomly assigned the same number of participants with more experience (over 5 years) and less experience (5 years or fewer) [6]. Randomisation was concealed, and participants were unaware of the resucitation scenario until the simulation began.

All participants signed an informed consent including permission to be recorded and to use images for data analysis, before being allocated into groups.

### Data Collection Procedure

On the day of the simulation each participant was required:

- To complete a survey collecting data regarding their demographic characteristics (professional experience, professional category, age, gender, type of hospital, regular use of drug-related apps at work, participation in simulations and the number of times nurses had to administer drugs in a pediatric CPR last year).
- Pre-briefing: Researchers did not have access to the allocation sequence prior to the participant’s inclusion in the study. After randomised allocation, participants watched a one-minute video (https://youtu.be/I5rTLegQ56Y?si=mdU7l1jAhyvQlebT) that explained the study and the materials to be used: simulation manikin (Resusci Baby QCPR, company: Laerdal medical, Norway), monitor screen, and materials for drug administration. In addition, if they were in the intervention group, they watched a five-minute video (https://youtu.be/WkGaT2T2RCg?si=e9_hKr4PCYZ8tIgk) on how to use the mobile app.
- Simulation: Each participant was exposed to a 10–15 min, fully video-recorded, standardised, realistic pediatric CPR scenario concerning a twelve-month-old child weighing 8 kg; the weight was told to each participant at the beginning of the resuscitation scenario. The simulation scenario was a closed room with a manikin, monitor screen, several drugs, and the necessary administration materials, with the participant wearing a high-definition camera on their head (GoPro HERO10 Black, company: GoPro, Inc, United States). They were asked to sequentially prepare and intravenously administer five drugs: epinephrine (0.01 mg/kg), sodium bicarbonate (1 mEq/kg), midazolam (0.2 mg/kg), norepinephrine (0.1 mcg/kg/min) and another norepinephrine dose (0.15 mcg/kg/min) with the support of the PEDIDOSIS app to assist in pediatric drug preparation or by following conventional pediatric drug preparation methods (such as printed or electronic medication tables, smartphones, calculators or no cognitive aid).
- Briefing: After the simulation, participants completed a 10-point Likert scale to measure their perceived satisfaction (“on a scale of 1 to 10, how satisfied were you with your preparation experience during CPR simulation?”) [32]. Perceived stress was assessed by self-assessment using a numerical 10-point Likert visual analogue scale (VAS) [33]. In the app group they completed a SUS (System Usability Scale) questionnaire. The SUS assesses effectiveness (i.e., the ability of users to use the product), efficiency (i.e., the effort required to use the product), and satisfaction (i.e., how users feel when using the product) [32]. It contains 10 questions with Likert-type responses from 1 to 5. A system with a score above 85 is considered to have excellent usability, while a system with a score between 68 and 84 has good usability [32, 34, 35].

Once the simulation was completed, two members of the research team independently reviewed the recorded videos. They independently filled out the data collection form hosted on REDCap. When discrepancies arose, both researchers reviewed the video where the discrepancy occurred. If they could not reach an agreement, a third member of the research team evaluated the case to reach a consensus. Due to the nature of the study, blinding of both the participants and the researchers was not feasible. Blinding was ensured for the data analysis. The statistical analysis was conducted by the Health Research Institute of Hospital Universitario La Paz (IdiPAZ).

### Outcomes

The primary outcome was the incidence of wrong dose administrations, defined as any deviation from the correct dose. The errors were further categorized based on their severity: defining wrong dose administration > 10% deviation from the correct dose [6, 17]; and large magnitude ≥ 25% deviation from the correct dose [6]; errors in each drug and number of ME per participant. Secondary outcomes were the elapsed time in seconds between the oral prescription by the physician and the time participants delivered the drug. Incorrect preparation was considered when there was a deviation from the maximum recommended concentration for the dilution of each drug. All outcomes were measured through videographic review.

### Statistical Analysis

Qualitative data were described using absolute frequencies and percentages, while quantitative data were presented with the mean and standard deviation or the median and interquartile range, depending on their distribution according to the Shapiro-Wilk test (for < 50 patients) or the Kolmogorov-Smirnov test (for ≥ 50 patients). For normal distributions (*p* > 0.05), the mean and standard deviation were used, and parametric tests for group comparisons were applied. For non-normal distributions (*p* ≤ 0.05), the median and interquartile range were used, with non-parametric tests for comparisons.

The Pearson Chi-square or Fisher’s exact test was used for comparing qualitative variables, with Fisher’s test applied for small samples or rare events. For the association between qualitative and quantitative variables, ANOVA or Kruskal-Wallis tests were employed, with equivalent Student’s t-tests or Mann-Whitney tests for two-category continuous variables. When multiple comparisons were performed, p-values were adjusted using the Bonferroni correction to control for the family-wise type I error rate. All statistical tests were two-tailed, with significance set at *p* < 0.05. Data were analysed using R version 4.3.3 (R Core Team, 2024).

## Results

### Demographics

**Fig. 2:**
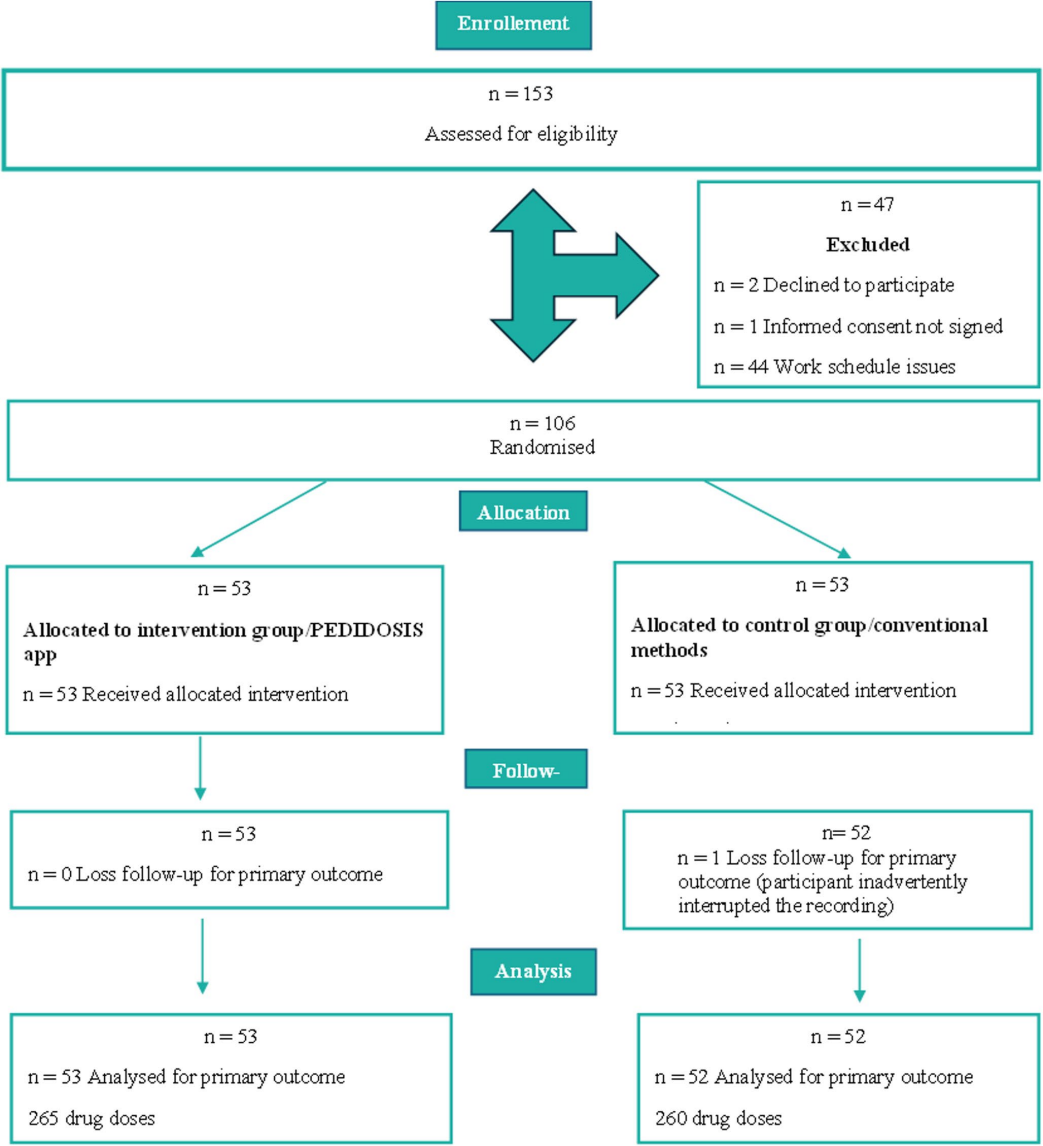
CONSORT Flow diagram of participants through the intervention [29]

A total of 153 nurses were invited to participate of whom 44 were excluded due to work schedule reasons, two did not wish to participate, and one did not sign the informed consent (Fig. 2, *Flow Diagram*). A total of 105 out of 106 (99.05%) participants completed the study, one participant was excluded from the final analysis because the video recording was accidentally stopped by the participant during the scenario. This resulted in 105 participants enrolled in the study, 38 (36.2%) with more than 5 years of work experience and 67 (63.8%) with less than 5 years. After enrolment and randomisation, 52 participants were assigned to the control group (conventional methods) and 53 to the intervention group (PEDIDOSIS app). In the control group, 18 participants (34.6%) had > 5 years of experience and 34 (65.4%) had ≤ 5 years. In the app group, these figures were 20 (37.7%) and 33 (62.3%), respectively. Each participant performed five drug administrations during the study, for a total of 265 drug doses in the PEDIDOSIS app group and 260 drug doses in the control group. Participants’ demographics are summarised in Table 1. Twenty participants (38.5%) in the conventional methods group compared to 33 (62.3%) in the app group reported that they had not administered any drugs during last year in a pediatric CPR situation (*p* < 0.05). No statistical difference was observed between groups among the rest of demographic characteristics.

**Table 1:** Participants demographics

Assigned group ^1^	Control group (conventional methods) (*n* = 52)	Intervention group (PEDIDOSIS app) (*n* = 53)	Total (*n* = 105)	*p* value ^2^
Years since nurse certification	8 [2.1, 18.3]	12 [3.0, 22.0]	10.2 [2.8, 21.0]	0.277
Professional experience in pediatrics (years)	2.0 [0.0–7.0]	2.0 [0.0–7.0]	2.0 [0.0–7.0]	0.601
Participant category				
Nurse	16 (30.8%)	27 (50.9%)	43 (41.0%)	0.106
Pediatric nurse specialist	21 (40.4%)	16 (30.2%)	37 (35.2%)	
Pediatric nursing resident	15 (28.8%)	10 (18.9%)	25 (23.8%)	
Age				
< 30 years	20 (38.5%)	18 (34.0%)	38 (36.2%)	0.503
30–39 years	14 (26.9%)	14 (26.4%)	28 (26.7%)	
40–49 years	13 (25.0%)	8 (15.1%)	21 (20.0%)	
≥ 50 years	5 (9.6%)	9 (17.0%)	14 (13.3%)	
Missing	0 (0.0%)	4 (7.5%)	4 (3.8%)	
Gender				
Woman	50 (96.2%)	49 (92.4%)	99 (94.3%)	1.000
Man	2 (3.8%)	3 (5.7%)	5 (4.8%)	
Missing	0 (0.0%)	1 (1.9%)	1 (0.9%)	
Type of hospital according to complexity				
High complexity	30 (57.7%)	31 (58.5%)	61 (58.1%)	0.934
Low complexity/monographic	22 (42.3%)	22 (41.5%)	44 (41.9%)	
Regular use of drug-related apps at work				
Almost never	4 (7.7%)	9 (17.0%)	13 (12.4%)	0.516
Occasionally	19 (36.5%)	14 (26.4%)	33 (31.4%)	
Usually	8 (15.4%)	11 (20.8%)	19 (18.1%)	
Almost always	16 (30.8%)	14 (26.4%)	30 (28.6%)	
Always	5 (9.6%)	5 (9.4%)	10 (9.5%)	
How many times have you had to administer drugs in a pediatric CPR situation in the last year? (number of times)				
0	20 (38.5%)	33 (62.3%)	53 (50.5%)	0.026*
1	18 (34.6%)	8 (15.1%)	26 (24.8%)	
> 1	9 (17.3%)	7 (13.2%)	16 (15.2%)	
Missing	5 (9.6%)	5 (9.4%)	10 (9.5%)	
Do you participate in clinical simulations at work?				
No	19 (36.5%)	13 (24.5%)	32 (30.5%)	0.181
Yes	33 (63.5%)	40 (75.5%)	73 (69.5%)	
Randomisation category				
Participant with ≤ 5 years’ experience	34 (65.4%)	33 (62.3%)	67 (63.8%)	0.739
Participant with > 5 years’ experience	18 (34.6%)	20 (37.7%)	38 (36.2%)	

^1^ Qualitative variables as numbers and percentages, n (%); quantitative variables as median and Interquartile range, median [Q1, Q3].

^2^ Kruskal-Wallis rank sum test for continuous outcomes / Pearson’s Chi-squared test and Fisher’s Exact Test for categorical outcomes.

* *p* < 0.05

### Primary Outcome

The absolute incidence of wrong dose administrations by any deviation from the correct dose was 134/260 (51.5%) for the control group vs. 70/265 (26.4%) for the PEDIDOSIS app group. Regarding > 10% wrong dose, the incidence was 94/260 (36.2%) in the control group and 41/265 (15.5%) in the app group.

Odds ratios compare PEDIDOSIS app vs. conventional methods, with values below 1, hence favoring PEDIDOSIS in terms of committing fewer administration errors. The OR for a wrong dose administration > 10% deviation with PEDIDOSIS app vs. conventional methods was 0.24 [0.14–0.4]), i.e., the risk of administering a > 10% wrong dose was significantly higher with conventional methods. Concretely, the risk difference was 20.7%. The OR for large magnitude ≥ 25% wrong dose administration with PEDIDOSIS app vs. conventional methods was 0.22 [0.09–0.49], i.e., the risk of administering largely wrong doses was higher with conventional methods. The risk difference was 12.8%.

The risk varied across drugs when using the conventional method, ranging from 34.6% for bicarbonate and midazolam to 76.9% for norepinephrine, whereas it was approximately 13.2%-47.2% for any drug when using the app (Table 2).

**Table 2:** Primary outcome. Number and proportion of medication errors

Variable	Control group (conventional methods)	Intervention group (PEDIDOSIS app)	Odds ratio ^2^ [95% CI]	*p* value	Risk difference (%)
Incorrect preparation	95/208 (45.7%)	45/212 (21.2%)	0.25 [0.14–0.43]	< 0.001***	24.5%
Medication error, wrong dose > 10%	94/260 (36.2%)	41/265 (15.5%)	0.24 [0.14–0.40]	< 0.001***	20.7%
Medication error, wrong dose, large magnitude ≥ 25%	48/260 (18.5%)	15/265 (5.7%)	0.22 [0.09–0.49]	< 0.001***	12.8%
Epinephrine, wrong dose ^1^	19/52 (36.5%)	7/53 (13.2%)	0.26 [0.10–0.70]	0.007**	23.3%
Bicarbonate, wrong dose ^1^	18/52 (34.6%)	4/53 (7.5%)	0.15 [0.05–0.50]	0.002**	27.1%
Midazolam, wrong dose ^1^	18/52 (34.6%)	10/53 (18.9%)	0.44 [0.18–1.07]	0.072	15.7%
Norepinephrine 0.1 mcg/kg/min, wrong dose ^1^	40/52 (76.9%)	24/53 (45.3%)	0.25 [0.11–0.58]	0.001**	31.6%
Norepinephrine 0.15 mcg/kg/min, wrong dose ^1^	40/52 (76.9%)	25/53 (47.2%)	0.3 [0.1–0.6]	0.002**	29.7%

^1^ Wrong dose: wrong dose administration defined as any deviation from the correct dose.

^2^ Odds ratios compare PEDIDOSIS app vs. conventional methods, with values below 1, hence favoring PEDIDOSIS in terms of commiting fewer administration errors

* *p* < 0.05 / ** *p* < 0.01 / *** *p* < 0.001

Of the participants using conventional methods, 9.6% did not commit any ME compared with 39.6% participants using the app. Moreover, the proportion of participants committing several ME (i.e., at least for 3 or more drugs) was substantially lower with the app 15.1% than with the conventional method 57.7% (Table 3). Additionally, percentage of > 10% wrong dose and large magnitude ME were lower with the app.

**Table 3:** Primary outcome. Number of medication errors per participant

Variables ^1^	Control group (conventional methods) (*n* = 52)	Intervention group (PEDIDOSIS app) (*n* = 53)	Total (*n* = 105)	*p* value ^2^
Total number of medication errors per participant				
0	5 (9.6%)	21 (39.6%)	26 (24.8%)	< 0.001***
1–2	17 (32.7%)	24 (45.3%)	41 (39.0%)	
3–5	30 (57.7%)	8 (15.1%)	38 (36.2%)	
Total number of medication errors > 10% per participant				
0	6 (11.5%)	22 (41.5%)	28 (26.7%)	< 0.001***
1	15 (28.8%)	22 (41.5%)	37 (35.2%)	
2	17 (32.7%)	8 (15.1%)	25 (23.8%)	
3	12 (23.1%)	1 (1.9%)	13 (12.4%)	
4	1 (1.9%)	0 (0.0%)	1 (1.0%)	
5	1 (1.9%)	0 (0.0%)	1 (1.0%)	
Total number of medication errors, large magnitude ≥ 25% per participant				
0	22 (42.3%)	42 (79.2%)	64 (61%)	0.001**
1	17 (32.7%)	7 (13.2%)	24 (22.9%)	
2	10 (19.2%)	4 (7.5%)	14 (13.3%)	
3	2 (3.8%)	0 (0%)	2 (1.9%)	
4	0 (0%)	0 (0%)	0 (0%)	
5	1 (1.9%)	0 (0%)	1 (1%)	

^1^ Qualitative variables as numbers and percentages, n (%)

^2^ Fisher’s Exact Test for Count Data for categorical outcomes.

* *p* < 0.05 / ** *p* < 0.01 / *** *p* < 0.001

### Secondary Outcomes

**Fig. 3:**
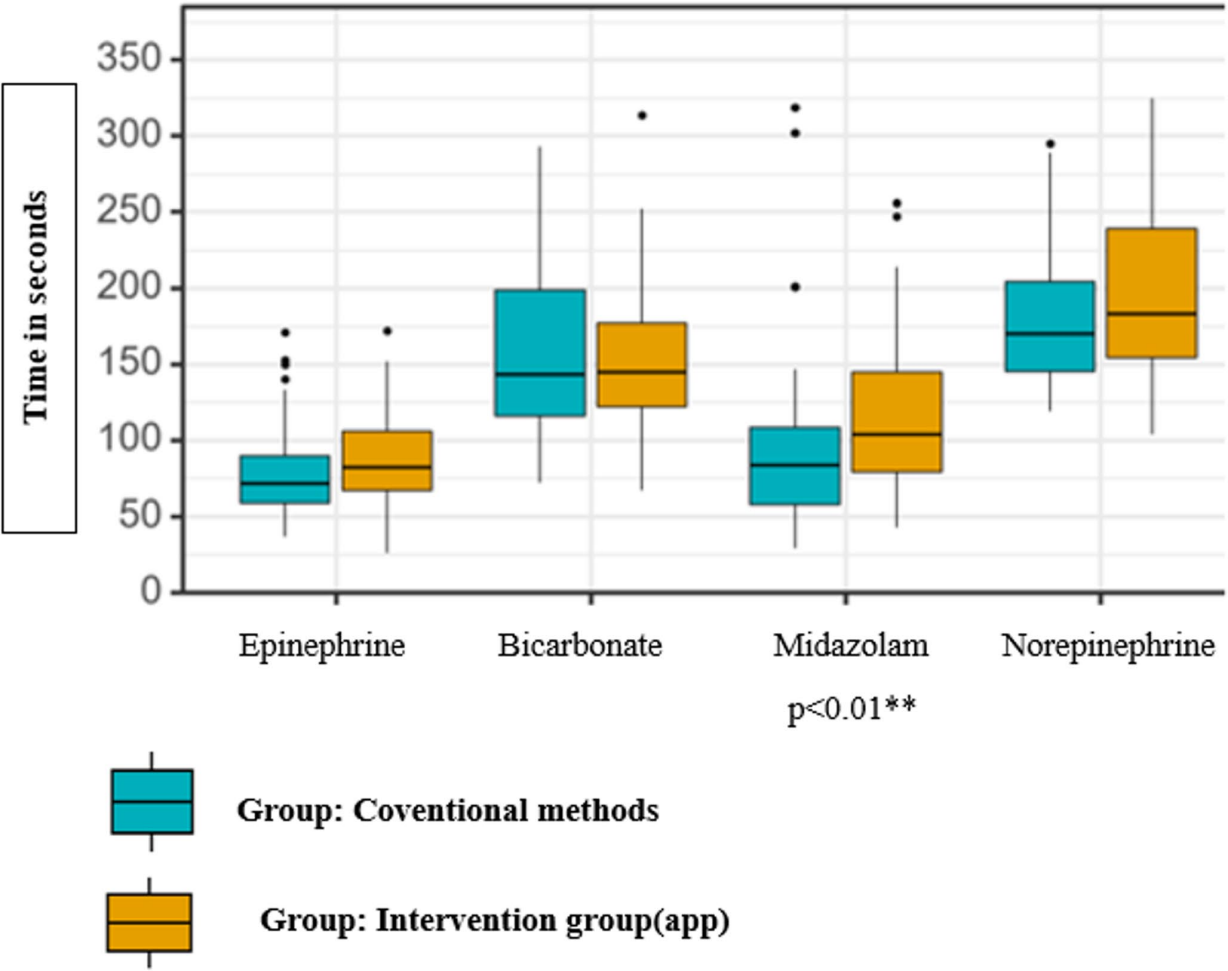
Time in seconds to drug administration. Elapsed time in seconds between the oral prescription by the physician and time to drug delivery by the participant. ** *p* < 0.01

The elapsed time in seconds between the oral prescription by the physician and the time participants delivered the drug was different for both groups: epinephrine, control group 71.5 (Q1: 59–Q3: 89.5) vs. app group 82 (Q1: 67–Q3: 106), *p* = 0.051; bicarbonate, control group 144.5 (Q1: 117–Q3: 205.75) vs. app group 145 (Q1: 122–Q3: 177), *p* = 0.863; midazolam, control group 83.5 (Q1: 57.75–Q3: 108.5) vs. app group 104 (Q1: 79–Q3: 145), *p* = 0.008; norepinephrine (0.1 µg/kg/min), control group 170 (Q1: 145–Q3: 204.5) vs. app group 184 (Q1: 155–Q3: 245), *p* = 0.067; norepinephrine (0.15 µg/kg/min), control group 5 (Q1: 4–Q3: 9) vs. app group 5 (Q1: 3–Q3: 8), *p* = 0.927 (Fig. 3). Shorter times for drug delivery were observed for four of the drugs when conventional methods were used. Statistically significant differences were only found for midazolam.

Table 4 describes the preparation of different drugs. In the administration of bicarbonate and epinephrine, preparation errors were higher in the conventional methods group. In the case of bicarbonate, of the 13 (25%) incorrect preparations, 9 (69.2%) were made below the minimum recommended concentration.

**Table 4:** Preparation of different drugs

Assigned group	Control group (conventional methods) (*n* = 52)	Intervention group (PEDIDOSIS app) (*n* = 53)	Total (*n* = 105)	*p* value^2^
Epinephrine				
Concentration mg/ml				0.313
Correct concentration (0.1 mg/ml)	39 (75.0%)	44 (83.0%)	83 (79.0%)	
Medication errors	13 (25.0%)	9 (17.0%)	22 (21.0%)	
Concentration error epinephrine				0.187
Concentration > 0.1 mg/ml	10 (76.9%)	4 (44.4%)	14 (63.6%)	
Concentration < 0.1 mg/ml	3 (23.1%)	5 (55.6%)	8 (36.4%)	
Bicarbonate				
Concentration mEq/ml				0.034*
Correct concentration (0.5 mEq/ml)	39 (75.0%)	48 (90.6%)	87 (82.9%)	
Medication errors	13 (25.0%)	5 (9.4%)	18 (17.1%)	
Concentration error bicarbonate				0.326
Concentration > 0.5 mEq/ml	4 (30.8%)	3 (60.0%)	7 (38.9%)	
Concentration < 0.5 mEq/ml	9 (69.2%)	2 (40.0%)	11 (61.1%)	
Norepinephrine				
Concentration mcg/ml ^3^				0.259
Median [Q1, Q3]	5.9 [5.9, 6]	6 [5.9, 6]	5.9 [5.9, 6]	
Norepinephrine infusion rate				0.495
Correct infusion rate (8 ml/h)	50 (96.2%)	53 (100.0%)	103 (98.1%)	
Medication errors	2 (3.8%)	0 (0.0%)	2 (1.9%)	

^1^ Qualitative variables as numbers and percentages, n (%); quantitative variables as median and Interquartile range, median [Q1, Q3].

^2^ Kruskal-Wallis rank sum test for continuous outcomes / Pearson’s Chi-squared test and Fisher’s Exact Test for categorical outcomes.

^3^ Noradrenaline: maximum concentration for peripheral venous access is 16–32 mcg/ml [36].

* *p* < 0.05

**Fig. 4:**
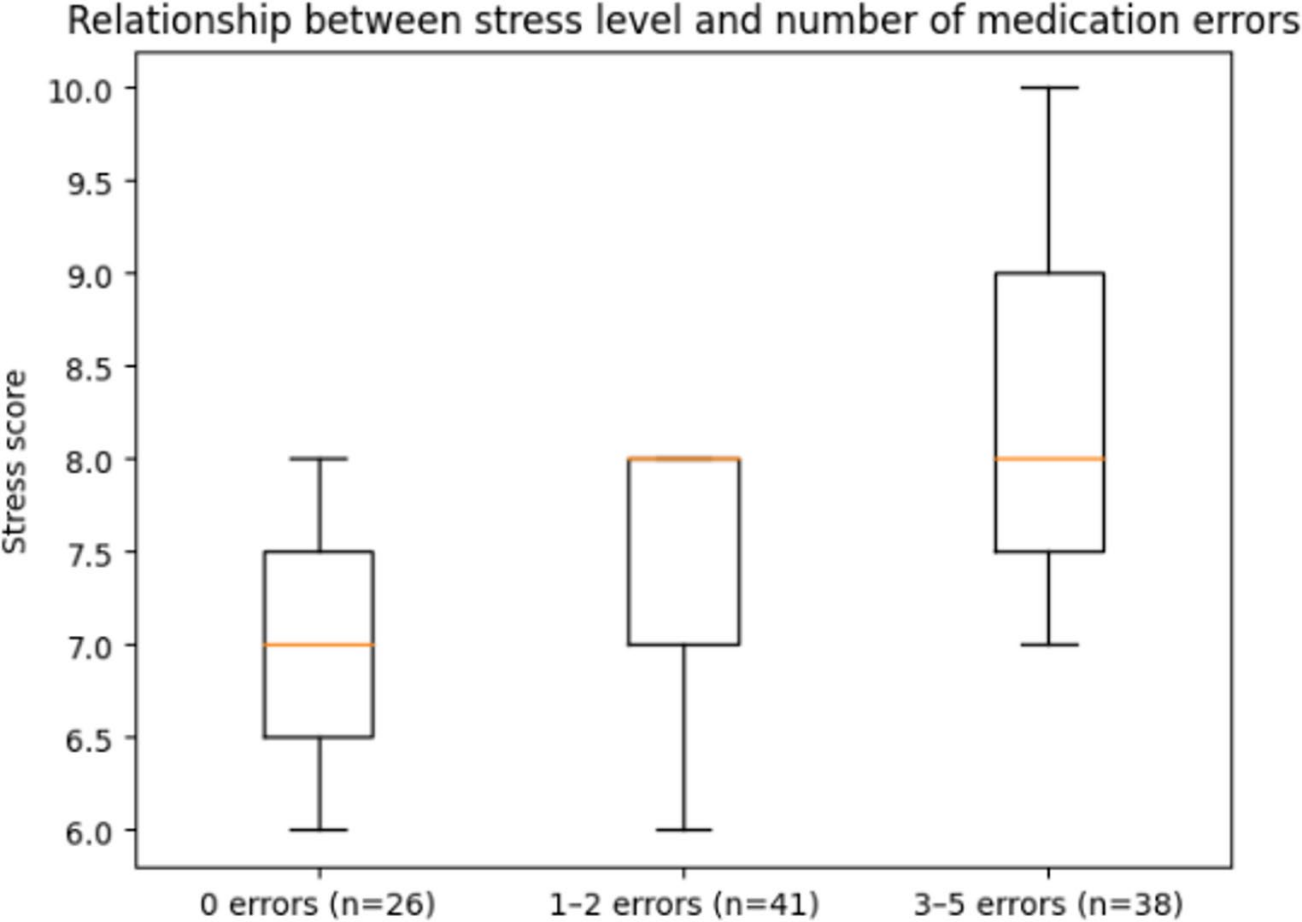
Relationship between stress levels (according to stress score) and number of medication errors

**Fig. 5:**
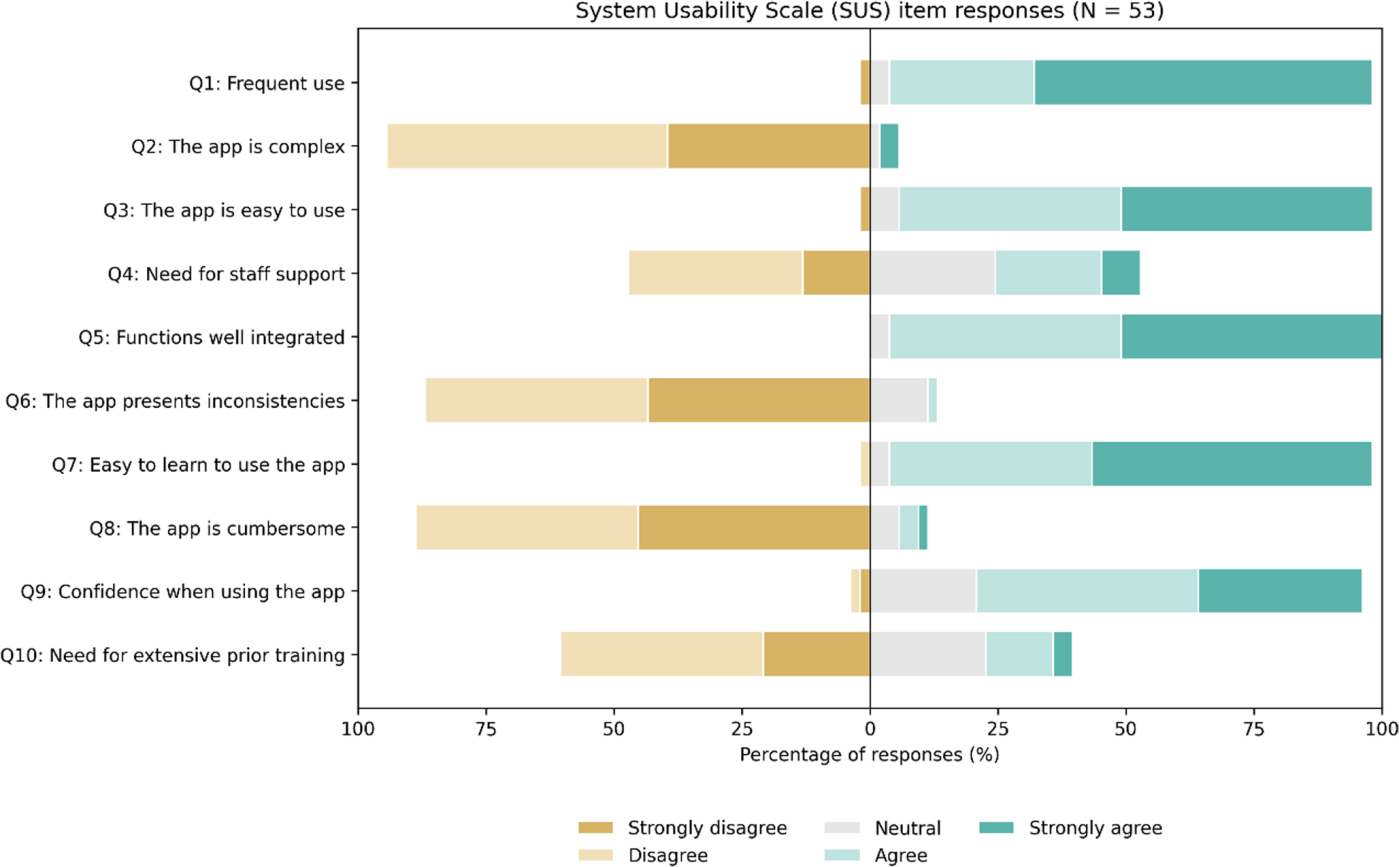
Distribution of responses to the System Usability Scale (SUS) items. Q1: I think that I would like to use this system frequently. Q2: I find the system unnecessarily complex. Q3: I think the system was easy to use. Q4: I think that I would need staff support to be able to use this system. Q5: I find that functions in this system are well integrated. Q6: I think there are too many inconsistencies in this system. Q7: I would imagine that most people would learn to use this system very quickly. Q8: I find the system very cumbersome to use. Q9: I felt very confident when using the system. Q10: I need extensive prior training before I could start using this system

Overall satisfaction regarding drug preparation was higher in the app group with a higher median of 7 (Q1:6-Q3:9) compared with the control group with a median of 6 (Q1:4-Q3:7). Stress levels showed a median of 7 (Q1:7-Q3:8) in the app group versus 8 (Q1:6.75-Q3:9.25). Figure 4 shows the relationship between stress level and number of ME. The SUS score obtained for PEDIDOSIS app was 78.92 out of 100, which is considered indicative of good usability. The distribution of responses to the SUS items (*n* = 53) is shown in Fig. 5.

## Discussion

In this multicentre randomised study, medication errors rates were significantly lower with the use of PEDIDOSIS app than with the use of conventional methods for administration of five drugs by nurses in a pediatric CPR simulation.

The most common type of ME in previous studies was incorrect dose, accounting for up to 65% of errors, with reported rates ranging from 26% to 70% in simulations [2, 4, 13]. In our study, error rates for bolus drugs (midazolam, epinephrine, bicarbonate) ranged from 21% to 26.7%, consistent with prior research [37, 38], while errors in norepinephrine infusions were markedly higher (76.9%), supporting the observation by Appelbaum et al. that dilution-prone drugs carry greater risk [6].

Errors in epinephrine, bicarbonate, and norepinephrine were significantly lower in the app group. The magnitude of reduction was similar to that reported by Siebert et al. for bolus drugs [5, 17] although in our study the absolute error rate for norepinephrine remained higher than in some previous reports [3, 13]. Overall, preparation errors were nearly halved in the app group (21.2% vs. 45.7%), confirming that the app substantially reduces, though does not eliminate, errors in complex preparations [13, 17]. Administration times were heterogeneous. The median time for epinephrine was 10.5 s longer with the app (82 vs. 71.5), aligning with Corazza et al. [14] but contrasting with studies that reported time savings [5, 17]. The mean time in both groups was close to the 79 s reported by Murugan [39], and the 10.5-second delay remains far below the one-minute interval known to affect survival (studies indicate that each minute of delay in epinephrine administration decreases survival by 9%) [4]. However, we recommend that a few additional seconds be devoted to confirming the correct dose if this ensures accurate administration which may ultimately be more favourable for patient outcomes in a real clinical setting. These discrepancies may also be attributed to variability in methodological design, differing digital software, or familiarity with the digital tool. In particular, the time to administer midazolam was shorter in conventional methods, but in this case, longer time in the app group is safer because the bolus should be administered in 2 min, as indicated by the app. Another study documented a saving of 54.9 s in the administration of norepinephrine with the app [3]. In our case, however, the results showed a longer administration time in the app group, as the median difference between groups was 14 s higher due to the app (with no statistical significance), although overall they took fewer seconds than in the reviewed studies.

The proportion of participants committing multiple errors (≥ 3) was substantially lower with the app (15.1% vs. 57.7%), a finding consistent with Siebert et al. (85.5% in the conventional group had ≥ 2 errors, compared to only 4.1% in the app group) [17].

Regarding stress, our study did not find statistically significant differences between groups, although a trend was observed linking higher stress levels with a greater number of errors. This aligns with AlGoraini et al. [40], who identified stress as a key factor in medication errors. While some studies have reported significant stress reduction with app use [13, 33], others have not [5]. In our sample, participants with no errors had a median stress score of 7, while those with 3–5 errors scored 8, although not statistically significant, it suggests potential clinical relevance. In the high-pressure context of a pediatric emergency, even modest reductions in stress could enhance perceived safety and professional confidence.

In relation to satisfaction perceived during the simulation, it was significantly higher in the app group. These results reinforce the idea that the incorporation of apps can enhance the experience of professionals in simulated clinical scenarios. These findings are consistent with previous research [13, 32]. Regarding usability, the score obtained in the SUS with PEDIDOSIS app corresponds to a usability considered good. Likewise, these results are in line with previous studies, such as PediAppRREST, which achieved 74.8, also within the good usability range [1], and pedAMINES, where even higher values were recorded 89.5 [32].

The PEDIDOSIS app is currently available in Spanish, and its drug library reflects Spanish pediatric emergency protocols; moreover, the content is periodically updated. International implementation would require localization (language adaptation and alignment with local drug concentrations and guidelines), supported by clinical validation and usability testing, and maintained through a governed update process to ensure patient safety across settings.

Recent medication-safety research is increasingly incorporating machine learning-based risk prediction and screening systems, supported by prospective direct-observation designs to identify real-world risk factors and improve prevention strategies in other pediatric settings [41, 42]. In this context, future iterations of PEDIDOSIS app could be integrated with an AI-based risk engine so that a predicted risk score (derived from patient or context variables and workflow signals) triggers adaptive decision support. For example, enhanced dose-check prompts or mandatory double-check steps only when risk is high. A prospective clinical evaluation incorporating direct observation would be an appropriate next step to validate this combined approach and strengthen external validity.

There are several limitations in this study. The main limitation lies in the simulated environment in which the research was conducted. Despite efforts to replicate a real-life situation, the simulation environment may not fully reflect the setting of a pediatric emergency. However, it is considered an appropriate environment for research, allowing the investigation of various issues that could not easily be standardised in a pediatric critical situation. Another limitation is that, in the intervention group, participants had only around three minutes to familiarise themselves with the app display, whereas in a hypothetical real-life situation, they would have had access to the app for several months. To avoid bias, the app was not provided in advance. There may also be a risk of selection bias, as the nursing staff are the ones who decide to participate voluntarily. The study was feasibility-driven and may be underpowered for smaller effects therefore results should be interpreted with confidence intervals. To mitigate the high risk of observer bias inherent in video-based assessment the first thing was that medication administration errors were coded using a pre-specified coding manual with operational definitions and a standardized data extraction form. Reviewers were trained using pilot videos and calibration meetings were held to ensure consistent interpretation of the criteria. Second, videos were not identified. Third, both reviewers assessed all videos independently, and when discrepancies arose a third researcher evaluated the case to reach a consensus among all researchers. Given the nature of the study, blinding could only be guaranteed for the personnel who performed the data analysis.

## Conclusion

PEDIDOSIS app reduced the error rate significantly compared to conventional preparation methods. Medication errors continue to be a significant challenge in healthcare, particularly in pediatric administrations. Additional research is required to assess their effectiveness in clinical settings.

## Data Availability

Available upon reasonable request.
